# Anesthetic efficacy of articaine/epinephrine plus mannitol in comparison with articaine/epinephrine anesthesia for inferior alveolar nerve block in patients with symptomatic irreversible pulpitis: A randomized controlled clinical trial

**DOI:** 10.15171/joddd.2019.048

**Published:** 2019

**Authors:** Sahar Shakoui, Mostafa Ghodrati, Negin Ghasemi, Tannaz Pourlak, Amir Ardalan Abdollahi

**Affiliations:** ^1^Oral and Periodontal Research Center, Tabriz University of Medical Sciences, Tabriz, Iran; ^2^Department of Periodontics, Faculty of Dentistry, Tabriz University of Medical Sciences, Tabriz, Iran; ^3^Department of Oral and Maxillofacial Surgery, Dental School, Tabriz University of Medical Sciences, Tabriz, Iran; ^4^Assistant Professor, Department of Endodontics, Dental School, Urmia University of Medical Sciences, Urmia, Iran.

**Keywords:** Articaine, irreversible pulpitis, mannitol

## Abstract

***Background.*** It is difficult to achieve successful pulpal anesthesia in mandibular posterior teeth with symptomatic irreversible pulpitis. The present study aimed to compare the effect of articaine/epinephrine anesthesia with articaine/epinephrine at a combination of 0.5 mol/mL of mannitol for the inferior alveolar nerve block (IANB) in patients presenting with symptomatic irreversible pulpitis in the mandibular first molar tooth.

***Methods.*** One hundred patients with symptomatic irreversible pulpitis in the mandibular first molar tooth were selected and randomly divided into two groups based on the injection method. The first group underwent an IANB technique with 1.8 mL of articaine, whereas the second group received 2.9 mL of a formulation, consisting of 1.8 mL of articaine plus 1.1 mL of 0.5 mol/L of mannitol. Fifteen minutes after injections and anesthesia of the lip, the access cavity was prepared. According to the visual analog scale (VAS) criteria, no pain or mild pain for caries removal, pulp exposure and canal instrumentation were regarded as success. Chi-squared test was used for the analysis of data. The level of significance was set at 0.05.

***Results.*** The success rate in the group with articaine/epinephrine anesthesia plus mannitol was higher than that in the group with articaine/epinephrine anesthesia, with no significant difference between the two groups (P>0.05).

***Conclusion.*** It was concluded, under the limitations of this study, that adding mannitol to articaine/epinephrine anesthesia did not increase the success of IANB in mandibular posterior teeth with symptomatic irreversible pulpitis.

## Introduction

Inferior alveolar nerve block (IANB) is the most common injection method used to induce anesthesia in mandibular posterior teeth for endodontic procedures.^1^ IANB is characterized by its significantly high failure rates, especially in patients presenting with symptomatic irreversible pulpitis.^2,3^ Endodontic studies on patients presenting with symptomatic irreversible pulpitis have reported failure rates from 44% to 81% for the IANB.^4,5^ This failure rate is associated with activated nociceptors in the inflamed pulp, leading to a lower pain threshold.^6,7^ Accordingly, recent studies have aimed to improve the success rate of IANB in patients presenting with symptomatic irreversible pulpitis using different injection methods, anesthetics solutions,^3,5^ complementary injection methods,^8,9^ drug therapy before IANB,^10,11^ and adding accessory substances to the formulations for local anesthetic agents.^12,13^

In this regard, mannitol has been recently used. Mannitol is a hexose sugar alcohol with a molecular weight of 182.17 g/mol. It can be naturally found in fruits and vegetables and is an osmotic diuretic.^14,15^ Since mannitol opens the membrane surrounding each nerve, it results in the penetration of more ions into the nerve.^16^ As a result, it might affect neural conduction and increase the success rate of IANB. Antonijevic et al^13^ concluded that 0.5 mol/mL of mannitol is very effective in opening the membrane surrounding each nerve, resulting in the penetration of more macromolecules and pumping of ions into the nerve. In addition, hyperosmolar solutions, like mannitol, have been shown to block the release of action potentials in type A neurons in mice.^17^

Articaine, after lidocaine, is the most widely used anesthetic agent in dentistry. Articaine is a dental amide-type local anesthetic agent, and instead of the benzene ring in lidocaine, it contains a thiophene ring, which increases its solubility in fat and accelerates its penetration through the membrane and bone.^18-20^ Compared with lidocaine, articaine induces faster and more durable anesthesia.^21-23^ Available studies indicate that articaine has equal effects in comparison with other local anesthetics in the IANB and maxillary infiltration; however, it is more successful than lidocaine in mandibular first molar infiltration technique.^24-27^

Recent studies have shown that mannitol plus lidocaine increases anesthesia in the IANB; however, there is a lack of studies on the effect of mannitol plus articaine on the induction of anesthesia in IANB in patients with symptomatic irreversible pulpitis. Therefore, this study investigated the efficacy of adding mannitol to articaine to increase the success rate of IANB in patients presenting with symptomatic irreversible pulpitis in the mandibular first molar teeth.

## Methods

The protocol of this study was approved by the Ethics Committee of Tabriz University of Medical Sciences (Ethics code: IR.TBZMED.REC.1396.1269) and registered at the Iranian Registry of Clinical Trials (Approval code: IRCT20180228038901N1).

The sample size was estimated at 50 samples in each group according to the results of a study by Kreimer et al,^10^ considering a success rate of 39% in the group with a combination of lidocaine/epinephrine and mannitol and 13% in the group with lidocaine/epinephrine with a difference of 26% in the success rate, α = 0.05, a study power of 80% and an error rate of 10%.

The current randomized controlled clinical trial was carried out on 100 patients referring to the Endodontic Department of Tabriz Faculty of Dentistry. The inclusion criteria consisted of systemically healthy patients, an age >18 years, no use of beta-blockers, no treatment with different types of opioids, no drug abuse, no pregnancy and lactation, no allergy to the materials used in the study, and no oral inflammation, or infection.

The patients included in this study had permanent mandibular first molar teeth with vital pulps and mature roots (confirmed by radiography), spontaneous pain, more severe and prolonged pain response, compared with the adjacent control tooth, to the cold testing with Endo-Ice ((1, 1, 1, 2 Tetrafluoroethane; Hygenic Crop., Akron, OH, USA) due to the decay, and normal periapical status. To avoid bias, the diagnostic steps were performed by an individual blinded to the injection of anesthetic solutions, and pain registration procedures.

The exclusion criteria of the study included patients with the following characteristics: no response to the cold test, teeth with widening of the periodontal ligament (PDL), and teeth with no vital coronal pulp tissue during access cavity preparation (partial necrosis).

One hundred patients, including 50 females and 50 males, were selected (n = 50 for each group) and randomly divided into two subgroups composed of 25 men and 25 women. The randomization was implemented with the Randlist software. A code from 1 to 50 (male and female) was assigned to each patient.

A standard injection of IANB, using conventional injection by a syringe with a 27-gauge needle with a length of 3.6 cm, was conducted in group one. The injection point was determined and aspiration was conducted, followed by the injection of 1.8 mL of 4% articaine solution with an epinephrine concentration of 1:200,000 (DENTACAIN, Exir, Tehran, Iran) in one minute.

The anesthetic solution for group two was prepared immediately before injection according to the following steps.

Under a sterile condition, 1.8 mL of 4% articaine solution with 1:200,000 epinephrine concentration (DENTACAIN, Exir, Tehran, Iran) from a standard dental cartridge plus 1.1 mL of 0.5 mol/L of 20% mannitol (200 mL) (Polifarma Pharmaceuticals Industry and Trade Co. Inc., Turkey) was taken into a 3-mL sterile Luer-lok tip syringe (AVAPEZESHK, Iran). The mixture was shaken 20 times to achieve a uniform mix. The mannitol solution was placed in water at 80°C before mixing for 15‒20 minutes to remove any crystals in the solution. The standard injection and the remaining steps were similar to those in the first group.

Fifteen minutes after injection, the patients were asked about lip anesthesia.^10^ Fourteen patients did not have lip anesthesia and were excluded, whereas 86 patients had lip anesthesia and were enrolled in the study.

The patients were informed about the stages of the study and informed consent was obtained. The teeth were isolated with a rubber dam and the access cavity was prepared. The patients were asked to raise their hand when they felt any pain and record their pain severity on VAS. Pain sensation steps were recorded separately using the following methods: upon dentin entry, upon the pulp chamber entry, and initial canal instrumentation.

The 170-mm Heft-Parker visual analog scale (VAS) was employed to assess pain,^10^ in which according to the location of pain, the pain level is classified into 4 categories: no pain (0 mm), mild pain (1‒54 mm), moderate pain (55‒112 mm), and severe pain (113‒170 mm).

Successful anesthesia was defined according to the VAS criteria representing no pain (0) or mild pain (1‒54 mm).

In this study, diagnostic tests of pulp status and IANB injection were performed by an operator, and the access cavity preparation and recording of the possible pain during operation were carried out by another operator.

### Statistical analysis 

Repeated-measures ANOVA was used to determine statistical differences in the means of pain intensity between the two groups through three stages of evaluation; chi-squared test was used to assess the relationship between gender and the study groups. The level of significance was defined at P<0.05.

## Results

**Figure 1:**
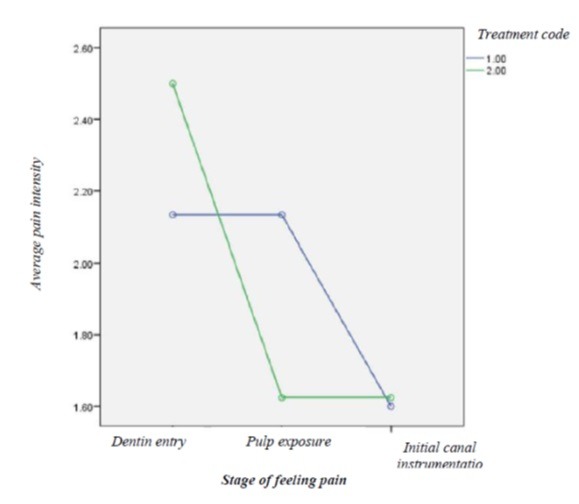
The linear chart for the mean of pain intensity in the studied groups.

The results of repeated-measures ANOVA showed no significant differences in the means of pain intensity between the two groups (P>0.05) (Table1). In addition, a significant difference was observed in the means of pain intensity between the three stages of evaluation (P<0.05) (Table 2, Figure 1).

**Table 1:** The results of repeated-measures ANOVA

**Variables**	**P-value**
**Group**	0.817
**Stage of evaluation**	0.002
**The relation between the stage and group**	0.078

P<0.05 was significant.

**Table 2:** The mean and standard deviation of the pain intensity in the studied groups

**Stage of feeling pain**	**Group**	**Mean**	**SD**
**Dentin entry**	Articaine	2.1333	0.35187
	Articaine/mannitol	2.5000	0.53452
	Total	2.2609	0.44898
**Pulp exposure**	Articaine	2.1333	0.83381
	Articaine/mannitol	1.6250	0.74402
	Total	1.9565	0.82453
**Initial canal instrumentation**	Articaine	1.6000	0.63246
	Articaine/mannitol	1.6250	0.51755
	Total	1.6087	0.58303

According to the results of chi-squared test, there was no statistically significant relationship between gender and the study groups (P>0.05).

## Discussion

Favorable pain control is essential in the treatment of painful endodontic diseases. The low success rate of IANB injection and poor control of pain in patients with irreversible pulpitis has resulted in an increase in the use of supplementary substances to increase the anesthetic success.^3,4^ The causative factors for the failure to achieve sufficient anesthesia in the IANB technique in the presence of inflammation include neurodegenerative changes throughout the inflamed nerve away from the inflammation site and the presence of amino acids produced by the lysosomal activity of proteolytic enzymes.^6,8^ Recent studies have assessed the use of auxiliary substances, such as meperidine, diphenhydramine, hyaluronidase, and sodium bicarbonate to enhance the success of the IANB technique. It has been indicated that none of these substances, compared with lidocaine/epinephrine, increase the success rate of IANB significantly.^12,28^

The failure of inferior alveolar anesthesia can be caused by the membrane surrounding the nerve, through which the anesthetic solution cannot fully penetrate to reach the nerve tract. It has been reported that mannitol increases the permeability of the membrane surrounding each nerve.^16^ Moreover, it changes or interrupts the action potential in neurons.^17^ Accordingly, in this study we studied the effect of mannitol. Studies have indicated that mannitol is a non-reactive substance,^15,16^ which is widely used in medicine. No study has yet compared the effect of articaine anesthetic solution with that of a combination of articaine and mannitol in patients with symptomatic irreversible pulpitis. In this regard, this study compared the success rate of articaine/mannitol with that of articaine for IANB injection in patients with symptomatic irreversible pulpitis. In the present study, the male and female patients were divided and analyzed equally, andsince the tooth type affects the IANB success rate, mandibular first molar teeth were selected. Gender and age of the patients did not result in a significant difference between the two study groups. In previous endodontic studies, IANB success rate was 19‒57% in patients with irreversible pulpitis.^3,5,10,12^ In this research, we used lip anesthesia as a clinical indicator of IANB success, ignoring the electric pulp testing (EPT) since Nasstein et al^29^ showed that in 42% of the cases which responded negatively to the EPT test, there was pain during endodontic procedures. Lip anesthesia does not guarantee a successful anesthesia of the pulp. Therefore, no pain (VAS = 0) or mild pain (VAS = 1‒54 mm) were considered as a success upon dentin entry, upon the pulp chamber entry, and initial canal instrumentation. The low success rate of articaine in the IANB technique for the patients presenting with symptomatic irreversible pulpitis was consistent with other studies.^30^ Since adequate pulpal anesthesia cannot be achieved by these formulations in patients with symptomatic irreversible pulpitis, supplementary injection techniques, such as intraosseous or intraligamentary, must be considered to provide favorable anesthesia.^2^ Further studies are recommended to increase the success rate of the IANB technique in patients with symptomatic irreversible pulpitis.

The results of this study showed that the articaine/epinephrine solution plus mannitol resulted in a higher success rate compared with articaine/epinephrine without mannitol; however, the difference was not statistically significant. Unlike the current study, Kreimer et al^10^ examined the effect of mannitol on the success rate of IANB for mandibular molars and showed significant differences in terms of success rate between the group with lidocaine/epinephrine plus mannitol and the group with lidocaine/epinephrine.It can be concluded that articaine itself has a high tissue and osseous penetration and diminishes the effect of mannitol, which increases the permeability of the nerve membrane.

## Conclusion

Under the limitations of this study, it was concluded that adding mannitol to articaine/epinephrine anesthetic agent did not increase the success rate of the IANB technique in mandibular posterior teeth with symptomatic irreversible pulpitis.

## Authors’ contributions

SS was responsible for the idea of the research, study design and observing the procedure of the study. MG was responsible for data collection and conducting the practical steps of the study. AAA carried out the data analysis and edited the manuscript. NG prepared the final manuscript.

## Acknowledgments

The authors wish to thank the Research Vice Chancellor and Dental and Periodontal Research Center of Tabriz University of Medical Sciences.

## Competing interests

None declared.

## Funding

Not applicable.

## Ethics approval

This study was approved by the Ethics Committee of Tabriz University of Medical Sciences (Ethics code: IR.TBZMED.REC.1396.1269) and registered at the Iranian Registry of Clinical Trials (Approval code: IRCT20180228038901N1).
